# Effects of Dietary Manganese Levels on Tibia Development, Bone Mineralization, and Cartilage Growth-Related Gene Expression in Squabs

**DOI:** 10.3390/ani16213524

**Published:** 2026-11-07

**Authors:** Jing Li, Lixin Liu, Chenlu Zhao, Haoxuan Li, Yuxin Shao, Yipu Li, Shuxian Xie, Asmaa Taha Yaseen Kishawy, Tenghe Ma, Zheng Wang, Bo Zhang

**Affiliations:** 1 Institute of Animal Husbandry and Veterinary Medicine, Beijing Academy of Agriculture and Forestry Sciences, Beijing 100097, China; xsu1980@163.com (J.L.); liulixinsun@hotmail.com (L.L.); zcl285196@163.com (C.Z.); crystalnihility@163.com (H.L.); syu123@sina.com (Y.S.); 18730978250@163.com (Y.L.); 17854271052@163.com (S.X.); 2 Department of Nutrition and Clinical Nutrition, Faculty of Veterinary Medicine, Zagazig University, Zagazig 44519, Egypt; no.dispair2000@gmail.com; 3 School of Life Science and Food Engineering, Hebei University of Science and Technology, Shijiazhuang 050091, China; matenghe@126.com

**Keywords:** pigeon, manganese, tibia development, bone quality, requirement

## Abstract

Manganese (Mn) is indispensable for skeletal development in poultry, yet its dietary requirement for meat pigeons has never been established. This study fed 120 pairs of White King pigeon breeders diets containing 28.43, 63.43, 98.43, 133.43, or 168.43 mg/kg total Mn (0, 35, 70, 105, or 140 mg/kg supplemental Mn) for 53 days to assess effects on reproductive performance, squab tibia development, and epiphyseal cartilage gene expression. Egg hatchability responded quadratically (*p* = 0.006), peaking at 97.9% in the 98.43 mg/kg group and falling to 83.3% at 168.43 mg/kg. Mn concentration in squab crop contents increased linearly with increasing dietary Mn levels provided to breeders (*p* = 0.009), indicating greater Mn exposure in the sampled offspring. However, this measurement does not directly quantify Mn transfer through pure crop milk. In 28-day-old squabs, serum alkaline phosphatase (ALP) activity was affected by dietary Mn level (overall treatment effect, *p* = 0.010) and showed a quadratic response (*p* = 0.003), with the highest activity observed in the 98.43 mg/kg total dietary Mn group. Tibial strength and maximum deflection did not differ among dietary treatments (*p* > 0.05). Tibial ash percentage showed a significant treatment effect (*p* = 0.001) and a linear response to dietary Mn level (*p* = 0.021), although the difference between the basal and highest-Mn groups was relatively modest (49.9% vs. 46.9%). These findings do not provide sufficient evidence of severe impairment of tibial mineralization at the highest dietary Mn level. In tibial epiphyseal cartilage, fibroblast growth factor 23 (*FGF23*) mRNA expression at 63.43 mg/kg total dietary Mn was approximately 3.85 times that in the basal group, whereas expression at 98.43~168.43 mg/kg did not differ significantly from the basal group. ETS-related gene (*ERG*) mRNA expression was approximately 5.63~5.85 times that in the basal group at 63.43~133.43 mg/kg, but expression at 168.43 mg/kg was lower than in these intermediate-dose groups and did not differ significantly from the basal group. Quadratic regression analyses based on replicate-level observations revealed significant relationships between dietary total Mn levels and egg hatchability (*R*^2^ = 0.931, *p* = 0.0155) and relative *ERG* expression (*R*^2^ = 0.783, *p* = 0.0368). The estimated optimal dietary total Mn levels were 92.63 and 95.34 mg/kg based on egg hatchability and ERG expression, respectively. These findings suggest a preliminary optimal dietary total Mn range of 92.63~95.34 mg/kg for meat pigeons under the conditions of this study, which requires further validation.

## Simple Summary

Manganese is an essential trace mineral for bone development and reproductive performance in poultry. However, the dietary manganese requirement of meat pigeons has not yet been established. In this study, White King pigeon breeders were fed diets containing different total Mn concentrations to evaluate reproductive performance, Mn exposure of squabs, and indicators of tibial development in 28-day-old squabs. Dietary Mn concentration was associated with changes in egg hatchability, serum alkaline phosphatase activity, selected tibial characteristics, and cartilage growth-related gene expression. Mn concentration in squab crop contents increased with dietary Mn concentration. Quadratic regression analyses based on replicate-level observations indicated estimated optimal total dietary Mn levels of 92.63 mg/kg based on egg hatchability and 95.34 mg/kg based on *ERG* expression. These results suggest a preliminary optimal dietary total Mn range of 92.63~95.34 mg/kg for meat pigeons under the conditions of this study. Further research is needed to validate this estimate.

## 1. Introduction

The pigeon (Columba livia) industry has emerged as the fourth largest poultry sector in China, following chickens, ducks, and geese [1,2]. Unlike these precocial species, pigeons are altricial birds with a distinct reproductive and rearing strategy. Squabs (young pigeons) lack the ability to forage independently from hatching until they reach market age at approximately 28 days [3,4]. During this rapid growth phase, they rely entirely on their parents for nourishment. In the first week after hatching, squabs are fed exclusively with pigeon milk, a secretion from the crop epithelium of both male and female breeders, and subsequently transition to a mixture of pigeon milk and partially digested feed [5,6]. This biological trait dictates that the nutritional intake of pigeon breeders fundamentally governs the skeletal and muscular development of squabs. However, the modernization of pigeon farming started relatively late, leaving a significant gap in standardized nutritional requirements. In commercial practice, pigeon diets are frequently formulated by borrowing nutritional standards from chickens or quails. Such practices overlook the dual nutritional burden placed on pigeon breeders, who must sustain their own reproductive functions while simultaneously meeting the intense developmental demands of their offspring.

Manganese (Mn) is an essential trace element that functions as a specific activator for glycosyltransferases and polysaccharide polymerases [7,8]. These enzymes are indispensable for the synthesis of chondroitin sulfate and other mucopolysaccharides within the bone organic matrix. In vertebrates, bone represents the primary Mn storage organ in the body [9,10]. In poultry, the tibia is particularly sensitive to dietary Mn status, as it serves as both a structural and mineral reservoir [11]. Consequently, bone development is highly sensitive to dietary Mn fluctuations. Previous studies in poultry have shown that inadequate Mn supply can impair endochondral ossification and contribute to skeletal abnormalities, such as perosis, shortened tibias, and enlarged joints [11,12,13]. Excessive Mn exposure has been associated with alterations in mineral metabolism and skeletal development in some animal models. However, the mechanisms underlying these effects and their relevance to meat pigeons at practical dietary Mn levels remain unclear [10,13].

While the Mn requirements for broilers (120 mg/kg) and laying hens (60 mg/kg) have been clearly defined by the National Standard of the P.R. China (NY/T 33-2004 and T/CAAA 038-2020 [14,15]), the optimal dietary Mn level for meat pigeons remains undetermined. Our preliminary analysis of an exploratory analytical screen of 12 commercially available pigeon-feed samples showed a wide range of measured total Mn concentrations (89.3~1440 mg/kg, Supplementary Table S1). This variation highlights the need for evidence-based, pigeon-specific Mn recommendations to guide diet formulation. Given that squabs achieve market weight in merely four weeks [16], their demand for trace elements to support rapid tibial elongation and mineralization is exceptionally high [17].

Endochondral ossification in the epiphyseal growth plate depends on coordinated chondrocyte proliferation, differentiation, hypertrophy, and matrix mineralization. In the present study, fibroblast growth factor 23 (*FGF23*) and ETS-related gene (*ERG*) were selected as mechanistically relevant candidate genes rather than as a comprehensive panel of conventional cartilage markers. *FGF23* is a key regulator of phosphate and vitamin D homeostasis and has been detected in growth plate chondrocytes, where it may contribute to the local regulation of chondrocyte differentiation and tissue mineralization. *ERG* is an ETS-family transcription factor implicated in chondrocyte differentiation and the maintenance of articular cartilage in mammalian systems. These genes were therefore used to explore mineralization-related and cartilage-regulatory pathways potentially associated with dietary Mn supply.

Therefore, this study was conducted to evaluate the effects of graded dietary Mn levels on the reproductive performance of pigeon breeders, as well as the tibia morphological characteristics, bone mineralization, and cartilage growth-related gene expression (*FGF23* and *ERG*) in squabs. By fitting quadratic regression models based on sensitive indicators, we aimed to estimate the optimal dietary Mn requirement for pigeons, providing a theoretical basis for precision feeding in the pigeon industry.

## 2. Materials and Methods

All animal care and experimental procedures in this study were approved by the Animal Care and Use Committee of the Beijing Academy of Agriculture and Forestry Sciences (Beijing, China, IAHVM11-2103-2).

### 2.1. Experimental Design, Animals, and Diets

A single-factor controlled feeding experiment was conducted. A total of 120 healthy, sexually mature White King pigeon breeding pairs (120 males and 120 females) with similar physiological status were assigned to five dietary treatments using a pre-specified cyclic allocation sequence. The five treatments were assigned repeatedly in an alternating order until 24 breeding-pair replicates had been allocated to each treatment. Thus, treatment allocation was systematic rather than based on a random sequence. Each breeding pair was housed in an individual cage and was considered the experimental unit for reproductive measurements. Each treatment consisted of 24 replicates. Each pair of breeding pigeons naturally incubates and feeds 2 squabs. The pre-experimental period lasted for 7 days, followed by an 18-day incubation period and a 28-day squab rearing period, totaling 53 days. The five experimental diets were prepared separately and were assigned to breeding pairs according to the cyclic treatment allocation. Each cage received only its assigned diet throughout the 53-day experimental period. Feed was provided separately to each cage, and no feed was shared among cages or treatments.

The basal diet was formulated according to the Technical Specification for Feeding and Management of Meat Pigeons (T/CAAA 038-2020 [15]). The composition and nutrient levels of the basal diet are shown in Table 1. The background Mn content in the basal diet was measured to be 28.43 mg/kg. Mn sulfate monohydrate (MnSO_4_·H_2_O, containing 32.51% Mn, purchased from Sinopharm Chemical Reagent Co., Ltd., Shanghai, China) was used as the exogenous Mn source. The 5 experimental diets were formulated by adding 0, 35, 70, 105, and 140 mg/kg of Mn to the basal diet. Therefore, the values of 28.43, 63.43, 98.43, 133.43, and 168.43 mg/kg represent calculated target total dietary Mn concentrations, derived from the measured basal-diet Mn concentration and the intended supplemental Mn concentrations. During the feed preparation process, the Mn source was first mixed with corn starch to create a core premix, which was then thoroughly mixed with other feed ingredients and pelleted into complete pelleted feed (4 mm in diameter and 10 mm in length).

### 2.2. Animal Housing and Management

The experiment was conducted at a commercial pigeon farm in Miyun District, Beijing, China. Each pair of breeding pigeons was housed in a separate plastic cage measuring 50 cm × 60 cm × 50 cm. The ambient temperature was maintained at 18~25 °C throughout the experiment. All experimental birds had ad libitum access to feed and water and were exposed to 18 h of light per day. The incubation of eggs and the feeding of squabs were performed naturally by the breeding pigeons. No cross-fostering was performed, and each breeding pair remained in its assigned cage and on its assigned dietary treatment throughout the experiment.

### 2.3. Sample Collection

At 28 days of age, terminal sampling was restricted to breeding pairs rearing two surviving squabs in order to standardize brood size and reduce variability associated with differences in offspring number. Eight eligible breeding-pair replicates were sampled from each dietary treatment. Within each sampled pair, one of the two squabs was randomly selected for blood collection and tibial sampling, resulting in eight sampled squabs per treatment (*n* = 8 per treatment). The sampled squabs were fasted for 6 h before blood collection. Blood samples (5 mL) were collected from the wing vein and centrifuged at 3000 r/min for 10 min at 24 °C. The separated serum was immediately frozen in liquid nitrogen and stored at −80 °C until analysis. Before laboratory analysis, samples were coded, and laboratory personnel were blinded to dietary treatment identity until the analytical results had been recorded.

After blood collection, the squabs were euthanized by carbon dioxide asphyxiation. Crop contents (squab crop contents) were collected from squabs and stored at −20 °C. The intact left and right tibias, including the epiphyseal cartilage, were rapidly dissected. The epiphyseal cartilage was carefully separated from the tibia, placed in sterile RNase-free tubes, snap-frozen in liquid nitrogen, and stored at −80 °C for gene expression analysis. The remaining tibial shafts were stripped of all adhering muscle and connective tissues, differentiated into left and right sides, and stored at −20 °C for subsequent physical and chemical analyses.

### 2.4. Determination of Mn Content in Squab Crop Contents

The squab crop contents samples were dried at 65 °C for 48 h. Approximately 0.5 g of the dried sample was placed in a 50 mL Erlenmeyer flask, and 9 mL of a digestion solution (nitric acid: perchloric acid = 5:1, *v*/*v*) was added. After pre-digestion at room temperature for 1 h, the mixture was heated on a hot plate at 300 °C until the solution became colorless and transparent. The final solution was diluted to 50 mL with deionized water, and the Mn concentration was determined using an inductively coupled plasma mass spectrometer (ICP-MS 7900, Agilent Technologies Inc., Santa Clara, CA, USA) [18,19].

### 2.5. Measurement of Serum Alkaline Phosphatase Activity

Serum alkaline phosphatase (ALP) activity was measured using an alkaline phosphatase (ALP/AKP) assay kit (microplate method; kit/catalogue no. A059-2-2, 96T; Nanjing Jiancheng Bioengineering Institute, Nanjing, China) according to the manufacturer’s instructions. According to the manufacturer’s instructions, the kit has a within-run coefficient of variation (CV) of ≤3%, a between-run CV of ≤5%, a recovery of 98%, and a linear range of 0–60 King units/100 mL. A reagent blank and the kit-supplied phenol standard were included in each assay plate according to the manufacturer’s protocol. Retain only if true: The blank absorbance value was required to be ≤0.150, and assays not meeting this criterion were repeated.

### 2.6. Evaluation of Tibia Morphological and Physical Characteristics

The left tibiae were thawed at 4 °C for morphological and physical measurements. Tibial length was measured as the distance between the midpoints of the proximal and distal ends using a digital caliper. Proximal mediolateral width and proximal anteroposterior thickness were measured at the proximal metaphysis, defined as approximately 25% of the total tibial length from the proximal end.

For the assessment of midshaft size, mediolateral width and anteroposterior thickness were measured separately at the midshaft, defined as 50% of the total tibial length from the proximal end [19,20]. Mean midshaft diameter was calculated as:

Mean midshaft diameter (cm) = (midshaft mediolateral width (cm) + midshaft anteroposterior thickness (cm))/2

Subsequently, the left tibiae were subjected to a three-point bending test using a HY-0580WEX mechanical testing machine (Hengyi, Shanghai, China). Each tibia was positioned on two supports with a span of 24 mm. A preload of 1 N was applied to establish stable contact between the tibia and the loading fixture. The loading head was positioned at the mid-diaphysis and advanced at a constant crosshead speed of 10 mm/min. The test was continued until tibial fracture occurred, which was defined as the failure criterion. Maximum force (N) and maximum deflection (mm) were recorded from the load–displacement curve.

### 2.7. Analysis of Tibia Chemical Composition

The right tibias were wiped dry and weighed to obtain the fresh weight. After drying at 105 °C for 3 h, the dry weight was recorded to calculate bone moisture content [21]. The dried tibias were then defatted using a Soxhlet extractor with low-temperature petroleum ether (boiling point 37 °C) in a 65 °C water bath for 24 h [22]. After solvent evaporation at 105 °C for 2 h, the defatted tibias were ashed in a muffle furnace at 600 °C for 12 h [23]. The ash weight was recorded to calculate bone ash percentage based on the corresponding gravimetric measurements.

The resulting bone ash was ground into a fine powder using a ceramic mortar. An accurately weighed portion of the bone ash powder was subjected to wet acid digestion using a mixture of nitric acid (HNO_3_) and perchloric acid (HClO_4_). Following digestion, the resulting solution was diluted to a defined final volume with deionized water.

The concentrations of calcium (Ca), phosphorus (P), and Mn were determined using inductively coupled plasma mass spectrometry (ICP-MS 7900, Agilent Technologies Inc., Santa Clara, CA, USA). Calibration curves were established using working standard solutions with known elemental concentrations after dilution.

Elemental concentrations in bone ash were calculated from the measured solution concentrations, final digestion volumes, applicable sample dilution factors, and accurately recorded bone ash sample masses. Ca and P concentrations were expressed as g/kg bone ash, whereas Mn concentration was expressed as mg/kg bone ash.

### 2.8. RNA Extraction and Quantitative Real-Time PCR

Total RNA was extracted from the epiphyseal cartilage of the tibia using Trizol reagent. The cartilage samples were ground in a liquid nitrogen-precooled ceramic mortar. The ground tissue was lysed with 500 μL TRIzol on ice for 30 min, followed by the addition of 100 μL chloroform. After centrifugation at 12,000 r/min for 20 min at 4 °C, the supernatant was mixed with an equal volume of isopropanol to precipitate the RNA. The RNA pellet was washed twice with 75% ethanol and dissolved in 20 μL of RNase-free water. The purity and concentration of the RNA were assessed using a microplate reader (OD260/OD280 ratio between 1.8 and 2.0).

The RNA was reverse-transcribed into cDNA using a reverse transcription kit (RR036A, TaKaRa, Shiga, Japan). Quantitative real-time PCR was performed using a fluorescence quantitative PCR kit (RR820A, TaKaRa) on a real-time PCR system (ABI 7500, Thermo Fisher Scientific, Inc., Waltham, MA, USA). The target genes included *CDC42*, *BCL2*, *BMP2*, *FGF23*, and *ERG*, with *GAPDH* serving as the internal reference gene. The primer sequences are listed in Table 2. Each sample was analyzed in triplicate. The relative mRNA expression levels of the target genes were calculated using the 2^−ΔΔCt^ method.

### 2.9. Statistical Analysis

All data were analyzed using SAS software version 9.4 (SAS Institute Inc., Cary, NC, USA). One-way analysis of variance (ANOVA) was performed to evaluate the main effects of dietary Mn levels. When significant differences were observed, means were separated using Duncan’s multiple comparisons. Orthogonal polynomial contrasts were used within the ANOVA framework to evaluate linear and quadratic trends in response to increasing dietary Mn levels. These trend analyses were performed separately from the quadratic regression analyses used for preliminary dietary Mn requirement estimation. Furthermore, the optimal dietary total Mn requirement for meat pigeons was estimated by fitting quadratic curve models using SAS 9.4 software based on sensitive indicators. Data are presented as mean ± standard deviation (SD). Statistical significance was defined as *p* < 0.05. The statistical significance and biological relevance of the fitted models were evaluated separately from the polynomial contrasts reported in the ANOVA tables.

To estimate the optimal dietary total Mn level, quadratic regression analyses were performed using replicate-level observations rather than treatment means. Egg hatchability was analyzed using data from 24 breeding-pair replicates per treatment (*n* = 120), whereas ERG relative mRNA expression was analyzed using data from eight replicates per treatment (*n* = 40). Dietary total Mn concentration was treated as a continuous independent variable, and the corresponding response variable was fitted to a quadratic model [24,25,26]. The model was formulated as follows:

*y* = *c* + *bx* + *ax*^2^

where *y* = egg hatchability or the relative mRNA expression of *ERG* in the epiphyseal cartilage of the tibia, *x* = dietary total Mn level (mg/kg), *c* = the intercept, *b* = the linear regression coefficient, and *a* = the quadratic regression coefficient. The optimal dietary total Mn level was estimated as the *x* value at the vertex of the quadratic curve, calculated as *x* = −*b*/(2*a*). The coefficient of determination (R^2^) and model *p*-value were used to evaluate model fit. Only statistically significant quadratic models with biologically interpretable responses and estimated vertices within the tested dietary Mn range were considered for requirement estimation.

## 3. Results

### 3.1. Effects of Dietary Mn Levels on Reproductive Performance of Pigeon Breeders

The effects of dietary Mn levels on the reproductive performance of pigeon breeders are presented in Table 3. Dietary Mn supplementation did not affect egg production or egg fertility (*p* > 0.05). For egg hatchability, the overall treatment effect did not reach statistical significance (*p* = 0.059), whereas the quadratic contrast was significant (*p* = 0.006).

### 3.2. Effects of Dietary Mn Levels on Mn Concentration in Squab Crop Contents and Serum ALP Activity of Squabs

The effects of dietary Mn levels on the Mn concentration in squab crop contents and serum alkaline phosphatase (ALP) activity of 28-day-old squabs are presented in Table 4. Dietary Mn supplementation significantly affected the Mn concentration in squab crop contents (*p* < 0.001), which showed a linear increase (*p* = 0.009) as dietary Mn levels rose from 28.43 to 168.43 mg/kg.

Dietary Mn levels also had a significant effect on serum ALP activity of squabs (*p* = 0.010). The serum ALP activity exhibited a significant quadratic response (*p* = 0.003) to increasing dietary Mn levels. The 98.43 mg/kg group had the highest serum ALP activity (550 ± 28.5 U/L), which was higher than that of all other treatment groups (*p* < 0.05).

### 3.3. Effects of Dietary Mn Levels on Tibia Morphological and Physical Characteristics of Squabs

The effects of dietary Mn levels on the tibia morphological and physical characteristics in 28-day-old squabs are presented in Table 4. Dietary Mn level did not affect tibial strength, maximum deflection, bone length, proximal mediolateral width, or proximal anteroposterior thickness (*p* > 0.05). However, the mean midshaft diameter differed significantly among dietary treatments (*p* = 0.018) and exhibited a significant quadratic response (*p* = 0.018). These results indicate that dietary Mn level influenced selected morphological characteristics of the tibia without detectable differences in the measured mechanical properties.

### 3.4. Effects of Dietary Mn Levels on Tibia Chemical Composition in Squabs

The effects of dietary Mn levels on the chemical composition of tibias from 28-day-old squabs are presented in Table 5. the Dietary Mn levels significantly affected tibial ash percentage (*p* = 0.001), Ca concentration (*p* = 0.001), *p* concentration (*p* = 0.008), and Mn concentration (*p* = 0.005), whereas the overall treatment effect on bone moisture content was not significant (*p* = 0.054). The tibial ash percentage differed significantly among dietary treatments (*p* = 0.001) and showed a significant linear contrast across dietary Mn levels (*p* = 0.021), although the observed group means did not follow a strictly monotonic pattern. The highest value was observed in the basal diet group (49.9%), whereas the lowest value occurred in the 63.43 mg/kg group (45.9%). No significant quadratic contrast was detected (*p* = 0.210). The Ca concentration in tibial ash exhibited significant linear (*p* = 0.006) and quadratic (*p* = 0.034) responses, with the highest concentration observed in the 168.43 mg/kg group (342 g/kg bone ash). In contrast, the *p* concentration in tibial ash showed a significant linear decrease (*p* = 0.002), from 171 g/kg bone ash in the basal diet group to 166 g/kg bone ash in the highest-Mn group. Across the five dietary treatments, the combined Ca and *p* concentrations accounted for approximately 49.9–50.8% of bone ash, with the Ca:*p* mass ratios ranging from approximately 1.95:1 to 2.06:1. The Mn concentration in tibial ash exhibited significant linear (*p* = 0.001) and quadratic (*p* < 0.001) responses, with the lowest concentration observed in the 63.43 mg/kg group (47.7 mg/kg bone ash) and the highest concentration in the 168.43 mg/kg group (63.0 mg/kg bone ash).

### 3.5. Effects of Dietary Mn Levels on Cartilage Growth-Related Gene Expression in the Epiphyseal Cartilage of Squab Tibia

The relative mRNA expression levels of cartilage growth-related genes in the epiphyseal cartilage of the tibia are presented in Table 6. The relative mRNA expression levels of *CDC42*, *BCL2*, and *BMP2* were not affected by dietary Mn levels (*p* > 0.05). Dietary Mn levels influenced the expression of *FGF23* (*p* = 0.009) and *ERG* (*p* = 0.003). The expression of *FGF23* showed a quadratic response (*p* = 0.013). Compared with the basal diet group, the 63.43 mg/kg group exhibited approximately 3.85-fold higher *FGF23* mRNA expression, which was greater than that of all other groups (*p* < 0.05). Similarly, the expression of *ERG* demonstrated a quadratic response (*p* = 0.001). The *ERG* mRNA expression was up-regulated by approximately 5.68-fold, 5.85-fold, and 5.63-fold in the 63.43, 98.43, and 133.43 mg/kg groups, respectively, compared with the basal diet group (*p* < 0.05). The 168.43 mg/kg group showed a reduction in *ERG* expression, declining to approximately 0.367-fold of the basal diet group level.

### 3.6. Estimation of Optimal Dietary Total Mn Requirement for Meat Pigeons

Quadratic regression was fitted to the treatment means of egg hatchability and relative *ERG* relative mRNA expression across the five pre-specified calculated target total dietary Mn levels. The quadratic regression analyses were conducted separately from the orthogonal polynomial contrasts presented in the ANOVA tables. Although several indicators, including serum ALP activity and tibial midshaft diameter, showed significant quadratic contrasts in the ANOVA, these results were not considered equivalent to the significance of the quadratic regression models used for dietary Mn requirement estimation. The selection of indicators for requirement estimation was based on the results of the separate regression analyses and the biological relevance of each response variable. Therefore, only egg hatchability and relative *ERG* mRNA expression were retained for preliminary dietary Mn requirement estimation.

To estimate the optimal dietary total Mn level, quadratic regression analyses were performed using replicate-level observations for egg hatchability and relative *ERG* relative mRNA expression. Both indicators exhibited statistically significant quadratic relationships with dietary total Mn levels. Egg hatchability showed a significant quadratic response (*y* = 74.88 + 0.4724*x* − 0.00255*x*^2^, *R*^2^ = 0.931, *p* = 0.0155), with an estimated optimal dietary total Mn level of 92.63 mg/kg. Similarly, the relative *ERG* mRNA expression in tibial epiphyseal cartilage showed a significant quadratic relationship with dietary total Mn levels (*y* = −4.955+ 0.2803*x* − 0.00147*x*^2^, *R*^2^ = 0.783, *p* = 0.0368), yielding an estimated optimal level of 95.34 mg/kg. Based on these two biologically relevant indicators, the estimated optimal range of total dietary Mn for meat pigeons was 92.63~95.34 mg/kg.

## 4. Discussion

### 4.1. Effects of Dietary Mn Levels on Reproductive Performance of Breeding Pigeons

Egg hatchability is a critical indicator of reproductive performance and economic efficiency in poultry breeders [27]. Previous studies have demonstrated that trace elements play a vital role in embryonic development and hatching success [28,29]. In the present study, the overall treatment test suggested a tendency for hatchability to differ among dietary Mn levels (*p* = 0.059), rather than a statistically significant overall effect at *p* < 0.05. This tendency was accompanied by a significant quadratic contrast (*p* = 0.006), with the highest observed mean of 97.9% ± 10.2% at 98.43 mg/kg total dietary Mn and lower means at the lowest and highest tested levels. This finding is consistent with previous research indicating that adequate Mn supplementation is essential for optimal reproductive performance in poultry [24]. Although the mean hatchability was numerically lower in the highest-Mn group than in the 98.43 mg/kg group, the overall treatment effect did not reach statistical significance (*p* = 0.059). Therefore, these findings do not establish a toxic effect of the highest tested dietary Mn level or interference with the absorption of other essential minerals.

### 4.2. Effects of Dietary Mn Levels on Squab Crop Contents, Serum ALP Activity, and Tibia Development of Squabs

Unlike other poultry species (precocial birds) that transfer trace elements to offspring primarily via the egg yolk, squabs (altricial birds) rely on squab crop contents as their main postnatal nutrient source [3,4,6]. In the present study, Mn concentration in squab crop contents increased linearly with increasing dietary Mn levels provided to breeding pigeons (*p* = 0.009). This finding indicates that higher dietary Mn supply to breeders was associated with increased Mn concentration in the crop contents of their offspring, suggesting greater Mn exposure in squabs. However, because crop contents were collected at 28 days of age and might have contained both crop milk and partially digested or regurgitated feed, these measurements did not directly quantify Mn transfer through pure crop milk. Nevertheless, the observed increase in crop-content Mn concentration is consistent with an association between maternal dietary Mn supply and Mn exposure in offspring.

ALP is primarily synthesized in the liver and bones. Bone-derived ALP, produced by osteoblasts, plays a crucial role in cellular material transport and bone mineralization, serving as an important indicator of osteoblast activity [30,31]. Research has shown that serum ALP activity in birds changes with age, and in young animals, the primary source of serum ALP is bone [32]. Therefore, high serum ALP activity reflects vigorous bone growth and development. In this study, the serum ALP activity of 28-day-old squabs exhibited a quadratic response to increasing dietary Mn levels, peaking in the 98.43 mg/kg group. This suggests that an appropriate level of dietary Mn can stimulate osteoblast activity and promote bone formation in squabs.

The tibia is a major weight-bearing bone, and its morphological and physical characteristics directly reflect the status of skeletal development [33]. In the present study, dietary Mn level significantly influenced tibial diameter, whereas tibial strength, maximum deflection, length, width, and thickness did not differ significantly among treatments. These findings suggest that dietary Mn may affect selected aspects of tibial morphology without producing detectable changes in the mechanical properties measured in this study. Therefore, the present results do not establish substantial impairment of tibial mechanical integrity at the highest tested dietary Mn level.

An important consideration when interpreting the present findings is that the Mn requirements of breeding pigeons and squabs may differ because of their distinct physiological stages and nutritional demands. Breeding pigeons require Mn to support maintenance and reproductive functions, whereas squabs have substantial nutritional demands associated with rapid post-hatch growth, cartilage development, and skeletal mineralization. In pigeons, squabs initially receive predominantly crop milk and subsequently transition to a mixture of crop milk and partially digested feed. Consequently, the dietary Mn concentration required to support the reproductive performance of breeding pigeons may not necessarily be identical to that required to optimize the growth and skeletal development of squabs. In the present study, Mn was supplemented to breeding pigeons, and the reproductive performance of breeders and developmental responses of their offspring were evaluated. Therefore, the estimated dietary Mn level should be interpreted as a preliminary estimate for the breeder diet under the conditions of this study, based on both reproductive and offspring-related responses, rather than as a directly established Mn requirement for squabs. Further studies involving direct dietary Mn supplementation of squabs at different post-hatch ages are needed to determine their specific Mn requirements.

### 4.3. Effects of Dietary Mn Levels on Tibia Composition in Squabs

Tibial ash content and mineral composition are commonly used to evaluate skeletal mineralization in poultry. However, these indicators should be interpreted together with bone morphological and mechanical characteristics, as changes in bone morphology do not necessarily correspond to changes in bone ash content or mechanical strength [33]. In the present study, dietary Mn levels significantly affected tibial ash percentage, with a significant overall linear contrast. However, the observed group means did not show a strictly monotonic response, as the lowest value occurred in the 63.43 mg/kg group rather than in the highest-Mn group. Therefore, the significant linear contrast should be interpreted as an statistical trend across dietary treatments rather than evidence of a continuous dose-dependent decrease in tibial ash percentage. Nevertheless, tibial strength and maximum deflection did not differ among treatments, suggesting that the observed changes in ash percentage should not be interpreted as evidence of severe impairment of bone mineralization.

Manganese plays an important role in skeletal development, particularly through its involvement in proteoglycan and glycosaminoglycan metabolism. Previous studies in poultry have shown that Mn availability influences the synthesis and composition of proteoglycans in avian growth plate cartilage [34,35]. These findings provide a biological basis for the involvement of Mn in skeletal matrix development. However, because the present study did not directly measure the bone organic matrix composition or proteoglycan content, the changes in tibial ash percentage cannot be attributed specifically to alterations in organic matrix formation.

Dietary Mn levels significantly affected the Ca, P, and Mn concentrations in tibial ash. Bone ash Ca concentration exhibited significant linear and quadratic responses, whereas P concentration showed a modest linear decrease. The combined Ca and P concentrations remained relatively stable across dietary treatments, accounting for approximately 49.9–50.8% of bone ash. Previous research in broiler chickens has also demonstrated that dietary Mn supplementation can influence tibial Ca concentration, although the responses of other mineral elements may differ [36].

Bone ash Mn concentration exhibited significant linear and quadratic responses to dietary Mn level, with the highest concentration observed in the 168.43 mg/kg group. Similarly, it has been reported that dietary Mn supplementation significantly influenced tibial Mn deposition in yellow-feathered broilers [37]. These findings indicate that bone Mn concentration is responsive to dietary Mn supply, although the response may not be strictly dose-dependent.

Importantly, the Ca, P, and Mn concentrations in the present study are expressed per unit mass of bone ash. Therefore, changes in their relative concentrations do not necessarily reflect corresponding changes in absolute mineral deposition or intestinal mineral absorption. Although dietary Mn levels affected tibial Ca and P concentrations, the combined Ca and P proportions in bone ash remained relatively stable (approximately 49.9–50.8%), with Ca mass ratios ranging from 1.95:1 to 2.06:1. Thus, the present findings do not provide direct evidence of a dietary Mn-induced Ca imbalance. It is possible that changes in dietary Mn supply may influence mineral homeostasis through alterations in intestinal mineral absorption, transport, or utilization. However, these mechanisms remain hypothetical because intestinal absorption, mineral transporter expression, and mineral excretion were not investigated in this study. Further studies incorporating these measurements are needed to clarify the mechanisms underlying the observed changes in skeletal mineral composition.

### 4.4. Effects of Dietary Mn Levels on Cartilage Growth-Related Gene Expression

The longitudinal growth of the tibia occurs primarily through endochondral ossification, a process in which the epiphyseal cartilage plays a central role [38]. This process involves the proliferation, differentiation, apoptosis, and mineralization of chondrocytes in the growth plate, regulated by various hormones and growth factors [38,39]. *FGF23* is a bone-derived hormone primarily secreted by osteocytes and osteoblasts, with additional expression detected in hypertrophic chondrocytes of the growth plate [40] and in immune cells under inflammatory conditions [41]. *FGF23* plays a central role in regulating phosphate homeostasis and vitamin D metabolism [42,43]. Although elevated *FGF23* has been shown to suppress osteoblast differentiation and matrix mineralization in vitro [44], the precise role of *FGF23* in the context of dietary Mn-regulated cartilage development remains to be fully characterized. *ERG* encodes a protein that acts as a transcriptional activator or repressor, regulating the development and differentiation of chondroprogenitor cells and playing a vital role in cell proliferation and apoptosis [45,46]. In our study, the relative mRNA expression levels of *FGF23* and *ERG* in the epiphyseal cartilage of squabs showed quadratic responses to dietary Mn levels. The expression of *FGF23* was up-regulated by approximately 3.85-fold in the 63.43 mg/kg group compared to the basal diet group. Similarly, *ERG* expression was significantly up-regulated by 5.63 to 5.85-fold in the 63.43, 98.43, and 133.43 mg/kg groups. These results suggest that dietary Mn levels may influence the expression of genes associated with cartilage growth and development in the epiphyseal cartilage of squabs. However, further studies incorporating histological and functional assessments are needed to determine whether these transcriptional changes translate into alterations in cartilage development. Conversely, the marked reduction in *ERG* expression in the 168.43 mg/kg group further supports the observation that excessive Mn is detrimental to cartilage development [10].

A further limitation is that conventional markers of chondrogenesis and growth plate maturation, including *SOX9*, *COL2A1*, *ACAN*, *COL10A1*, and *RUNX2*, were not measured. Future studies integrating these markers with histological assessment of the growth plate are needed to more comprehensively define the effects of dietary Mn on cartilage development.

### 4.5. Estimation of Optimal Dietary Total Mn Requirement

Determining the optimal dietary requirements for trace elements is essential for maximizing animal performance while minimizing environmental excretion and potential toxicity [47]. In the present study, the optimal dietary total Mn level was estimated using quadratic regression analyses based on replicate-level observations of two biologically relevant indicators: egg hatchability and *ERG* mRNA expression in tibial epiphyseal cartilage. This approach retained the variation among experimental replicates rather than relying solely on treatment means.

Both indicators exhibited significant quadratic relationships with dietary total Mn levels. The estimated optimal level was 92.63 mg/kg based on egg hatchability and 95.34 mg/kg based on *ERG* expression. The relatively close estimates obtained from these two independent biological indicators suggest that a dietary total Mn level of 92.63~95.34 mg/kg may be appropriate under the conditions of the present study.

Nevertheless, these estimates should be interpreted within the context of the experimental conditions and the biological indicators evaluated. Egg hatchability reflects reproductive performance, whereas *ERG* expression provides molecular information related to cartilage development. Neither indicator on its own can fully represent the overall physiological Mn requirement. Therefore, the estimated range should be considered a preliminary reference for dietary Mn formulation in meat pigeons, and further studies involving additional reproductive, skeletal, and physiological indicators are needed to validate this estimate.

Although the present study provides preliminary estimates of dietary Mn levels associated with reproductive and offspring-related responses, these estimates require further validation. Independent dose response trials incorporating additional Mn concentrations, particularly within and around the estimated range, are needed to confirm the dose response relationships and refine the estimated optimal level. Moreover, future analyses should use individual-level data and appropriate statistical models that account for the experimental unit and potential clustering effects, such as the nesting of squabs within breeder pairs or cages. These approaches will help improve the accuracy and generalizability of dietary Mn requirement estimates.

## 5. Conclusions

In conclusion, dietary Mn supplementation significantly impacts or shows influential trends with egg hatchability, squab crop contents Mn concentration, serum ALP activity, tibia morphological and physical characteristics, tibia chemical composition, and the mRNA expression of *FGF23* and *ERG* in the epiphyseal cartilage of squabs. Dietary Mn level was found to be associated with changes in serum ALP activity, tibial diameter, tibial ash percentage, and the expression of cartilage growth-related genes in squabs. No significant differences in tibial strength or maximum deflection were detected among treatments. The present findings do not provide sufficient evidence to conclude that the highest tested dietary Mn level severely impaired tibial mineralization. Based on quadratic regression analyses using replicate-level observations, the estimated optimal dietary total Mn levels were 92.63 mg/kg for egg hatchability and 95.34 mg/kg for *ERG* mRNA expression. These estimates suggest a preliminary optimal dietary total Mn range of 92.63~95.34 mg/kg for meat pigeons under the experimental conditions of this study. Further investigations incorporating additional biological indicators are required to validate this estimated range.

## Figures and Tables

**Table 1:** Composition and nutrient levels of the basal diet (air-dry basis).

Ingredient	Content (1000 kg)
Corn (kg)	410.0
Soybean meal (44% CP, kg)	131.3
Pea (kg)	174.2
Wheat (kg)	128.0
Cornstarch (kg)	2.460
Sorghum (kg)	104.0
Soybean oil (kg)	7.00
Dicalcium phosphate (kg)	20.87
Limestone (kg)	13.50
Sodium chloride (kg)	4.00
Vitamin premix ^1^ (kg)	0.300
Choline chloride (kg)	0.400
Mineral premix ^2^ (kg)	1.12
DL-Methionine (kg)	1.30
Lysine hydrochloride (kg)	1.60
Calculated composition	
Metabolizable energy, Mcal/kg	2.84
Crude protein (%)	14.60
Calcium (%)	1.11
Total phosphorus	0.74
Non-phytate phosphorus (available phosphorus, %)	0.44
Lysine (%)	0.88
Methionine (%)	0.36
Methionine + cysteine (%)	0.50
Threonine (%)	0.53
Mn ^3^, mg/kg	28.43

^1^ Complex vitamins are provided per kilogram of feed: VA 13,500 IU, VD3 3600 IU, VE 36 IU, VK3 4.5 mg, VB1 3.6 mg, VB2 11.25 mg, VB3 39 mg, VB5 16.5 mg, VB6 6 mg, VB12 0.039 mg, folic acid 2.1 mg and biotin 0.24 mg. ^2^ Trace components are provided per kilogram of feed: iron 150 mg, zinc 70 mg, copper 8 mg, iodine 0.35 mg, selenium 0.25 mg. ^3^ The value was detected by ICP-MS, and the other parameters in Table 1 are calculated values.

**Table 2:** Primer Sequence Listing.

Gene	Genebank Number	Primer Sequence (5′–3′)	Production Length
*GAPDH*	NM_001282835.1	Forward: GTCAGCAATGCCTCTTGCAC	105 bp
		Reverse: GATGGCATGGACAGTGGTCA	
*CDC42*	XM_005512394.2	Forward: GCCAAGAACAAGCAGAAGCC	189 bp
		Reverse: TCATAGCAGCACACACCTGC	
*BCL2*	XM_005509733.3	Forward: TGGCCTTCTTCGAGTTTGGT	183 bp
		Reverse: AAGGCCTCATACTGTTGCCA	
*BMP2*	XM_021294346.1	Forward: AGCACGGCGCTTCTTCTTTA	107 bp
		Reverse: TGTTCTCTGAGGTTCCGTGC	
*FGF23*	XM_005503041.2	Forward: AACGCTGATGGCTACGTTGA	125 bp
		Reverse: TCCATGCACAGATAGCGTCC	
*ERG*	XM_005511356.2	Forward: TCCAGCAGCTCACATCAAGG	120 bp
		Reverse: TCACTGGAGGAAGAGGCTGT	

**Table 3:** Effects of Dietary Mn levels on reproduction performance of pigeon breeders.

Item	Dietary Mn Content (mg/kg)					*p*-Value		
	28.43	63.43	98.43	133.43	168.43	Mn	Mn Linear	Mn Quadratic
Egg production (%)	89.6 ± 20.7	91.7 ± 19.0	91.7 ± 19.0	89.6 ± 20.7	89.6 ± 20.7	0.989	0.978	0.684
Egg fertility (%)	89.6 ± 20.7	91.7 ± 19.0	89.6 ± 20.7	95.8 ± 14.1	93.8 ± 16.9	0.722	0.895	1.00
Egg hatchability (%)	85.4 ± 27.5 ^ab^	95.8 ± 14.1 ^ab^	97.9 ± 10.2 ^a^	89.6 ± 20.7 ^ab^	83.3 ± 24.0 ^b^	0.059	0.673	0.006

The results were mean ± standard deviation (SD), with 24 replicates per group (*n* = 24). a–b: different letter marks represent significant differences (*p* < 0.05).

**Table 4:** Effects of Dietary Mn levels on tibia development and tibia quality of 28-day-old squabs.

Item	Dietary Mn Content (mg/kg)					*p*-Value		
	28.43	63.43	98.43	133.43	168.43	Mn	Mn Linear	Mn Quadratic
Mn concentration in squab crop contents (mg/kg)	12.3 ± 3.29 ^a^	12.9 ± 1.32 ^a^	29.0 ± 8.16 ^b^	35.1 ± 8.47 ^b^	41.6 ± 5.02 ^c^	<0.001	0.009	0.838
Serum ALP (U/L)	325 ± 50.5 ^b^	350 ± 18.2 ^b^	550 ± 28.5 ^a^	376 ± 48.7 ^b^	374 ± 20.0 ^b^	0.010	0.278	0.003
Bone strength (N)	63.4 ± 7.14	66.7 ± 10.9	58.9 ± 5.44	64.16 ± 3.97	63.1 ± 9.59	0.585	0.598	0.637
Maximum Deflection (mm)	2.47 ± 0.441	2.13 ± 0.247	2.29 ± 0.124	2.50 ± 0.178	2.36 ± 0.211	0.199	0.462	0.511
Bone length (cm)	5.97 ± 0.298	5.93 ± 0.238	6.05 ± 0.305	6.06 ± 0.238	6.00 ± 0.338	0.871	0.654	0.212
Proximal mediolateral width (cm)	0.599 ± 0.0497	0.638 ± 0.0496	0.602 ± 0.0488	0.608 ± 0.0197	0.566 ± 0.0395	0.109	0.199	0.087
Proximal anteroposterior thickness (cm)	0.419 ± 0.0477	0.438 ± 0.0486	0.426 ± 0.0268	0.422 ± 0.0187	0.395 ± 0.0199	0.272	0.321	0.156
Mean midshaft diameter (cm)	0.373 ± 0.0297 ^b^	0.388 ± 0.0198 ^a^	0.380 ± 0.011 ^ab^	0.389 ± 0.0102 ^a^	0.381 ± 0.0100 ^ab^	0.018	0.334	0.018

a–c: different letter marks represent significant differences (*p* < 0.05). The results were mean ± standard deviation (SD), with 8 replicates per group (*n* = 8).

**Table 5:** Effects of dietary Mn levels on tibia components of 28-day-old squabs.

Item	Dietary Mn Content (mg/kg)					*p*-Value		
	28.43	63.43	98.43	133.43	168.43	Mn	Mn Linear	Mn Quadratic
Bone moisture content (%)	24.3 ± 1.99	30.0 ± 2.23	31.0 ± 1.58	23.2 ± 2.56	22.5 ± 3.27	0.054	0.125	0.022
Bone ash content (%)	49.9 ± 0.592 ^a^	45.9 ± 0.585 ^c^	48.8 ± 0.763 ^ab^	47.0 ± 0.543 ^bc^	46.9 ± 0.622 ^bc^	0.001	0.021	0.210
Bone Ca content (g/kg bone ash)	334 ± 2.62 ^b^	331 ± 1.62 ^c^	334 ± 0.782 ^b^	338 ± 1.01 ^ab^	342 ± 1.54 ^a^	0.001	0.006	0.034
Bone P content (g/kg bone ash)	171 ± 1.58 ^a^	168 ± 0.858 ^ab^	168 ± 0.755 ^ab^	169 ± 0.845 ^ab^	166 ± 0.444 ^b^	0.008	0.002	0.849
Bone Mn content (mg/kg bone ash)	53.8 ± 1.14 ^b^	47.7 ± 1.91 ^c^	51.5 ± 2.89 ^b^	51.6 ± 1.48 ^b^	63.0 ± 1.35 ^a^	0.005	0.001	<0.001

a–c: different letter marks represent significant differences (*p* < 0.05). The results were mean ± standard deviation (SD), with 8 replicates per group (*n* = 8).

**Table 6:** Effects of dietary Mn level on gene expression in epiphyseal cartilage of tibia of 28-day-old squabs.

Item	Dietary Mn Content (mg/kg)					*p*-Value		
	28.43	63.43	98.43	133.43	168.43	Mn	Mn-Linear	Mn-Quadratic
*CDC42*	0.972 ± 0.464	0.779 ± 0.212	1.10 ± 0.902	0.881 ± 0.210	0.941 ± 0.213	0.848	0.895	0.899
*BCL2*	1.07 ± 0.422	2.20 ± 1.88	1.79 ± 1.25	0.923 ± 0.289	0.931 ± 0.182	0.139	0.235	0.011
*BMP2*	0.911 ± 0.271	4.59 ± 5.15	4.14 ± 4.43	4.82 ± 7.37	0.628 ± 0.251	0.262	0.342	0.277
*FGF23*	0.852 ± 0.263 ^b^	3.28 ± 1.20 ^a^	1.00 ± 0.82 ^b^	1.48 ± 0.90 ^b^	0.730 ± 0.438 ^b^	0.009	0.073	0.013
*ERG*	0.987 ± 0.535 ^b^	5.61 ± 1.35 ^a^	5.77 ± 1.33 ^a^	5.56 ± 2.23 ^a^	0.362 ± 0.121 ^b^	0.003	0.526	0.001

a–b: different letter marks represent significant differences (*p* < 0.05). The results were mean ± standard deviation (SD), with 8 replicates per group (*n* = 8).

## Data Availability

Data are contained within the article.

## Supplementary Materials

The following supporting information can be downloaded at: https://www.mdpi.com/article/10.3390/ani16213524/s1, Table S1: An exploratory analytical screen of 12 commercially available pigeon-feed samples.
