# Supplementary material for: Effects of Dietary Manganese Levels on Tibia Development, Bone Mineralization, and Cartilage Growth-Related Gene Expression in Squabs
Source: Animals (Basel). 2026 Nov 7;16(21):3524. doi: 10.3390/ani16213524 (PMC13761491; doi:10.3390/ani16213524)
Supplement: Supplementary file 1 [file animals-16-03524-s001.zip › animals-4627403-supplementary.pdf]

Supplementary Table S1. An exploratory analytical screen of 12 commercially available pigeon-feed samples

City	Mn(mg/kg)
Shandong	124.4
Shijiazhuang	141.3
Panjin	217.9
shannxi	446.2
Sichuan	109.5
Benxi	89.3
Changsha	94.4
Xingtai	154.6
Lingshou	812.6
Rizhao	949
Huizhou	1440
Guangzhou	1001.4
